# Training in oncoplastic surgery for mastologists

**DOI:** 10.1590/1806-9282.2024S119

**Published:** 2024-06-07

**Authors:** Augusto Tufi Hassan, Cicero de Andrade Urban, Gil Facina, Ruffo Freitas-Junior, Regis Resende Paulinelli, Jorge Villanova Biazus, Vilmar Marques de Oliveira, Rene Aloisio da Costa Vieira

**Affiliations:** 1 Brazilian Society of Mastology, Executive Board – Rio de Janeiro (RJ), Brazil.; 2 Clínica de Assistência à Mulher – Salvador (BA), Brazil.; 3 Brazilian Society of Mastology, Department of Oncoplastic Surgery – Rio de Janeiro (RJ), Brazil.; 4 Hospital Nossa Senhora da Graça – Curitiba (PR), Brazil.; 5 Universidade Federal de São Paulo – São Paulo (SP), Brazil.; 6 Universidade Federal de Goiás, Complexo Oncológico de Referência do Estado de Goiás, Advanced Center for Diagnosis of Breast Disease – Goiânia (GO), Brazil.; 7 Hospital de Câncer Araújo Jorge, Associação de Combate ao Câncer de Goiás – Goiânia (GO), Brazil.; 8 Hospital das Clínicas de Porto Alegre – Porto Alegre (RS), Brazil.; 9 Santa Casa de Misericórdia de São Paulo – São Paulo (SP), Brazil.; 10 Hospital de Câncer de Muriaé – Muriaé (MG), Brazil.

**Keywords:** Segmental mastectomy, Mammaplasty, Oncoplastic breast surgery, Medical education, Fellowships and scholarships

## Abstract

**OBJECTIVE:**

The radical change in the treatment of breast cancer has promoted the necessity for more comprehensive training of the professionals involved, ensuring the preservation of oncological safety while also allowing for cosmetic interventions to benefit breast cancer survivors. The aim of this study was to present the methods employed in the training of breast surgeons, highlighting the importance of oncoplasty and breast reconstruction.

**METHODS:**

A literature review was conducted in two databases, identifying articles related to medical education in the context of oncoplastic surgery and breast reconstruction. We also assessed the Brazilian experience in oncoplastic centers.

**RESULTS:**

The basis for educational discussions was derived from 16 articles. We observed approaches that included hands-on courses utilizing simulator models, porcine models, cadaver labs, and fellowship programs. Positive outcomes were observed in Brazil, a fact based on seven oncoplasty training centers for senior mastologists and five training centers for junior mastologists. From 2009 to 2023, an estimated 452 seniors and 42 juniors received training, representing approximately 30% of mastologists in Brazil who have acquired training and experience in oncoplasty.

**CONCLUSION:**

Despite the limited number of publications on training methods, oncoplastic centers have made significant progress in Brazil, establishing a successful model that can be replicated in other countries.

## INTRODUCTION

The surgical treatment of breast cancer has undergone a radical transformation in recent years. We have transitioned from radical mastectomies to breast-conserving therapy (BCT), which has been demonstrated to be as safe as radical surgery^1,2^ in the long term. Initially, conservative treatment was indicated for tumors up to 3 cm in size, with subsequent expansion to 5 cm and a favorable breast-to-tumor ratio. Simultaneously, for invasive carcinomas, the ideal margin changed from 1 cm to the absence of tumor at the inked margin^3^.

In the beginning, patients who underwent mastectomies often underwent delayed breast reconstructions with myocutaneous flaps, and now we perform immediate reconstructions. Implants typically used for breast augmentation became an integral part of immediate reconstruction^4^, facilitated by techniques such as skin and nipple-sparing mastectomies^5^. These reconstructions, traditionally performed by plastic surgeons, have also become part of the skill set of breast surgeons.

Concerning BCT, the need for tactics to preserve the breast and avoid unsatisfactory outcomes presented a challenge. The concept of oncoplastic breast surgery (OBS) emerged nearly a decade ago, initially met with resistance, but is now widely accepted by breast surgeons^6-9^. Techniques have been categorized based on breast location^10,11^, multicentricity/multifocality, and breast-to-tumor ratio^12^. Current literature shows that oncoplastic surgery (OPS) is safe, has acceptable recurrence rates, and is associated with improved cosmetic outcomes and greater patient satisfaction^13,14^.

As the paradigm shifted, it became necessary to prepare breast surgeons for breast or skin preservation, focusing on cosmetic quality and achieving acceptable local recurrence rates without compromising survival. Senior breast surgeons needed to enhance their skills. Although the literature on training methodologies is limited, this challenge was initially discussed only as a perspective^8^. In 2009, the first international consensus on this subject was established^15^. There are reports of hands-on courses on OPS^16^, simulator models^17,18^, porcine models^19^, and cadaver labs^20,21^. For the new breast surgeon, fellowship programs in OPS were also established^22,23^.

In Brazil and other countries, the advancement of mastology as a specialty has contributed to significant progress in surgical techniques. This has complemented existing techniques and fostered the substantial development of oncoplasty and breast reconstruction^24^. The concept of OPS has been embraced by the Brazilian Society of Mastology, leading to the creation of training courses for senior mastologists^20,21^. These courses have taken various formats, including biennial, annual, or modular. Consequently, the Brazilian Oncoplasty Journey, a national symposium organized by the Brazilian Society of Mastology, was established. Additionally, there has been a notable increase in the number of fellows in OPS. Simultaneously, OPS has been recognized as a necessary component of mastology medical residency programs throughout Brazil.

However, the literature on experiences with education in oncoplasty is limited^25^. Despite witnessing a quantitative growth in the number of mastologists performing OPS in Brazil, much of this experience remains undocumented. There is a shortage of studies discussing OPS training in both Brazil and abroad, a gap that justifies the present study.

## METHODS

This study is a systematic integrative review designed to analyze training methodologies in OPS for breast surgeons. To identify relevant literature, the PICO methodology was employed, with the following components: P=breast reconstruction or OPS or oncoplasty; I=medical education or fellowship; C=all articles; and O=all articles.

For keyword selection, two databases, PubMed and Lilacs, were utilized. The chosen keywords were drawn from Mesh terms or words deemed relevant to the study. No language restrictions were imposed, and the search was extended until September 30, 2023. In PubMed, the following search query was applied: ("Mammaplasty" [Mesh] OR "Mastectomy, Segmental" [Mesh] OR "oncoplastic surgery" OR "oncoplasty" OR "oncoplastic breast surgery") AND ("Education, Medical" [Mesh] OR "Fellowships and Scholarships" [Mesh]). In Lilacs, the following query was employed: "Educação Medica" (subject descriptor) and "neoplasias da mama" (subject descriptor).

**Figure 1:**
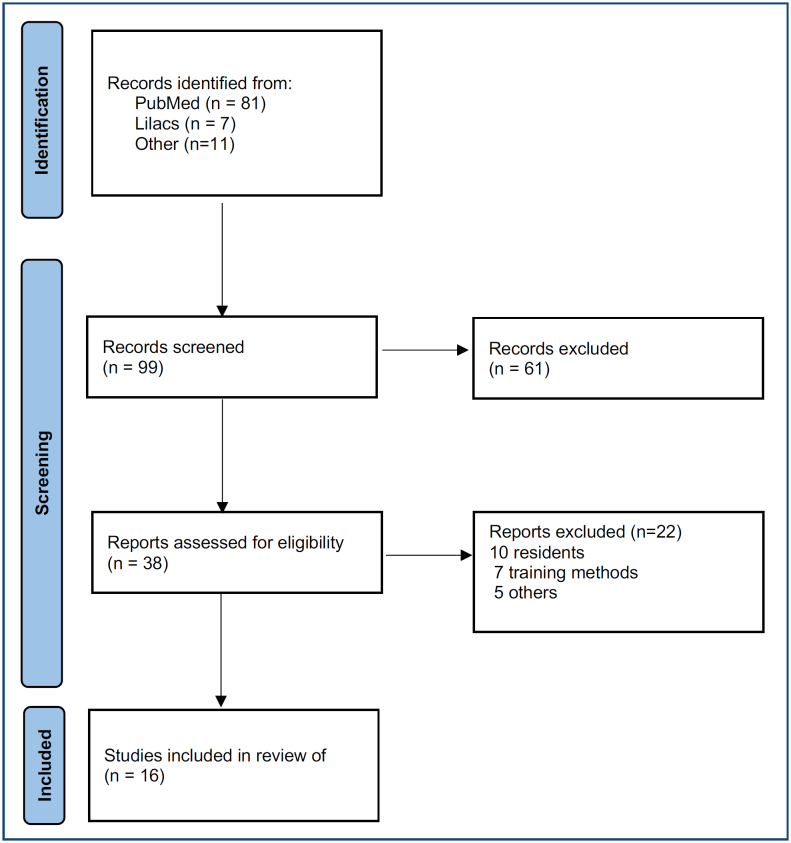
PRISMA flowchart.

Following the initial search, articles were selected based on their titles and abstracts. Selected articles were then obtained in full and evaluated for their relevance to the study's focus. In cases where there were multiple publications from the same research group addressing the same topic, the most recent publication was included. Figure 1 illustrates the application of the PRISMA methodology in article selection. To improve the information on oncoplasty training in Brazil, records from the Brazilian Society of Mastology and information obtained from Training Center Coordinators were examined. This examination aimed to provide a retrospective analysis of oncoplasty training for both senior mastologists (Table 1) and junior mastologists (Table 2). Information was directly collected from the training centers.

**Table 1:** Oncoplastic training centers in Brazil for senior mastologists.

Course	H.C. Porto Alegre	H.C. Barretos^20^	S.C.M. de São Paulo^21^	S.C.M. de Belo Horizonte^27^	H.C. Araújo Jorge^27^	H.C. Amaral Carvalho	H.C. Pernambuco
Location	Porto Alegre/RS	Barretos/SP	São Paulo/SP	Belo Horizonte/MG	Goiania/GO	Jaú/SP	Recife/PE
Beginning	2008	2009	2010	2011	2014	2016	2023
End	Current	2018	Current	2018	Current	Current	Current
Format	Theoretical-practical	Theoretical-practical	Theoretical-practical	Theoretical-practical	Theoretical-practical	Theoretical-practical	Theoretical-practical
Format	Modular	Continuous	Continuous	Continuous	Continuous	Continuous	Continuous
Duration	4 modules/course	21 months	10 months	9–15 months	11 months	11 months	12 months
Total number of hours/month	40 h/module	12–20 h	20 h	36 h	25 h	20 h	30 h
Total hours	160/year	252–420	200	320–540	240–275	220	360
Attendees/class	46 modules	12	10	12	12	12	12
Classes	2008–2019 2021–2023	5	13	5	9	7	1
Graduated*	13 years/4 modules/4 students	60	130	60	108	60	–
Current face-to-face attendees	4×4	Finished in 2018	Paused in 2023	Finished in 2018	12	12	12
Observation	Modular/thematic	8 h: 2009 20 h: 2015	Beneficência Portuguesa		2022: 15 online 2023: 23 online		

H.C.: Hospital do Câncer (Cancer Hospital); S.C.M.: Santa Casa de Misericórdia (Holy House of Mercy).

* Estimation.

**Table 2:** Oncoplastic training centers in Brazil for junior mastologists.

Course	H.C. Araújo Jorge	S.C.M. de Belo Horizonte	H.C. Barretos	H.G. de Fortaleza	H.G. in Universidade de Caxias
Location	Goiânia/GO	Belo Horizonte/MG	Barretos/SP	Fortaleza/CE	Caxias do Sul/RS
Beginning	2014	2015	2016	2017	2023
End	Current	Current	Current	Paused	Current
Residency in hospital	Mastology/surgical oncology	Mastology/plastic surgery	Mastology/surgical oncology	Mastology/plastic surgery	Mastology
Attendees/year	1	1–3	2	2	1
Activities	Breast reconstruction	Mastology/breast reconstruction	Mastology/breast reconstruction	Mastology/breast reconstruction	Mastology/breast reconstruction
Frequency	Annual	Annual	Annual	Annual	Annual
Certification	Fellow	Fellow	Lato-sensu in oncology	Fellow	Fellow
No. of graduated	9	7	13	7	0
No. of current attendees	1	3	1	0	1

H.C.: Hospital do Câncer (Cancer Hospital); S.C.M.: Santa Casa de Misericórdia (Holy House of Mercy); H.G.: Hospital Geral (General Hospital).

This study reports a literature review and uses publicly available data. In accordance with Resolution 466/2012 of the National Research Ethics Committee (CONEP) in Brazil, this research does not require evaluation by an Ethics Committee.

## RESULTS

Using the PRISMA methodology, we initially identified 88 studies, with 81 coming from PubMed and 7 from LILACS. To expand the dataset, 11 additional studies were incorporated, resulting in a total of 99 studies. No duplicate articles were encountered. Applying the selection criteria, 16 articles were ultimately chosen for comprehensive examination, representing the primary focus of this article. These articles were categorized as follows: consensus^15^, hands-on OPS courses^16^, simulators^17,18,26^, porcine models^19^, or practical courses in humans/cadaver labs^20,21^, fellows in OPS^22,23,27,28^, medical residency^29,30^, learning curve^31^, and limitations and perspectives^32^.

Regarding the oncoplasty training centers for senior mastologists in Brazil, we identified a total of seven centers, two of which had published their results^20,21^. In the context of training senior mastologists (Table 1), seven courses were conducted in Brazil. The first course started in 2008, and at present, five courses remain ongoing. These programs are distributed across five capital cities and two medium-sized city in Interior of São Paulo State. Additionally, five of them are held in cancer hospitals, while the other two take place in general hospitals. Six of these programs feature monthly meetings, with the number of total hours varying from 200 h to 540 h. A modular course format was also observed, featuring four independent modules: (1) oncological mammoplasty; (2) myocutaneous flaps and fat grafting; (3) reconstruction with implants and fat grafting; and (4) fat grafting in conservative surgery and refinements. In 2022, one of the courses adopted a dual-track format, offering both face-to-face and virtual sessions, thereby accommodating participants from numerous countries and providing simultaneous translation into Portuguese, English, and Spanish (www.oncoplasticsurgerycourse.com). Adding up all the attendees, besides those who started in 2023, we will have about 452 students who completed the course.

Assessing the training of junior mastologists (fellowship program) who have recently concluded their residencies, we identified five training centers (Table 2). These centers run annual programs located in hospitals that offer medical residency programs in mastology, surgical oncology, or plastic surgery. Among these centers, three are situated in capital cities, while two are affiliated with cancer hospitals. Including all the centers that started in 2023, there will be 42 graduates.

## DISCUSSION

In the 1990s, Audretsch coined the term "oncoplasty"^27^ to describe a new approach to breast cancer surgery that combined oncological principles with plastic surgery techniques. However, it was not until 2003 that a publication discussing the importance of training breast surgeons in reconstructive procedures^8^ was observed. In 2007, EUSOMA recommended that breast surgeons receive training in OPS^33^. In 2009, there was a consensus on oncoplastic training, emphasizing the need for collaboration between plastic surgeons and breast surgeons in various scenarios and the accreditation of training centers^15^.

Audretsch used the term "oncoplasty" as a synonym for tumor-specific breast reconstruction, so it was used as associated with reconstruction after mastectomy and BCT^15^. AndradeUrban^9^ proposed three levels of competencies for OPS: Level I for basic procedures that do not require specific training in plastic surgery; Level II for mastopexy, breast augmentation, lipofilling, Grisotti flap, reconstruction with implants, and bilateral procedures; and Level III for complex procedures with flaps. Clough et al.^10^ introduced a classification based on resection volume, distinguishing between Level I (resections less than 20% of breast volume) and Level II (extensive resections, representing 20–50% of breast volume). In 2019, a consensus by the American Society of Breast Surgeons^7,11^ introduced the terms "volume replacement" and "volume displacement." In the volume replacement group, techniques such as Level I (<20% volume excision) and Level II (20–50% techniques) were included. Techniques for volume replacement (>50%) include local/regional flaps, myocutaneous flaps, and implant-based reconstruction. This categorization is crucial for evaluating publications related to OPS training.

A study compared surgeons who performed oncoplastic procedures with those who did not. Factors associated with the use of oncoplastic techniques included male sex, fewer years of practice (<10 years), previous training in oncoplasty, and greater availability of plastic surgeons. Surprisingly, plastic surgeons were less related to breast preservation studies than oncoplastic breast surgeons^34^. Questionnaires were applied to surgeons who participated in an oncoplastic course in Australia and New Zealand that lasted two years, consisting of monthly classes. For the 59% (33/56) respondents, cost and time constraints were identified as negative factors affecting course participation^35^. Several barriers to surgeon training in oncoplasty were observed, including a lack of time to access oncoplastic educational material or courses^36^ the lengthy training period for breast surgeons, the non-recognition of the breast sub-specialty in some countries, and the necessity for dual (oncological and reconstructive) training^32^.

One of the great problems with different learning models is the establishment of methodologies for evaluating learning outcomes, such as knowledge or skill retention. Therefore, in order to evaluate potential methodologies that can assist in OPS training, we observed the results in the simulators made in Montreal^17^ in a hands-on course held in Canada^16^. This study compared senior and junior surgeons’ skills in the procedure of subpectoral breast augmentations^17^. They concluded that a hands-on course helps surgeons adopt OPS in their clinical practice^17^. A randomized study conducted in Singapore compared the performance of OPS performed on humans and in simulators. It was noted that although surgeons initially showed superior knowledge using the simulator, the results were similar^26^ after six months.

There is no defined minimum number of procedures to achieve expertise in oncoplasty. The British Association of Surgical Oncology suggested a minimum number of procedures for oncoplasty, with 25 for Level I and 50 for Level II^37^. The regional Australian experience was published in 2012, showing quantitative data in which the fellow performed 91 procedures as the first surgeon and 73 as an assistant^22^. A retrospective study evaluating the learning curve observed that 58 procedures were needed to reduce surgical time^31^.

In England, training courses in cadaver labs were started, and oncoplasty became a sub-specialty after breast surgery or plastic surgery^33^. In 2002, an investment was made in nine centers, creating 100 training and qualification scholarships for fellows for a 12-month period^27,28^. They selected breast or plastic surgeons with a minimum of 15 years of training in breast surgery, as they were considered for breast center accreditation^33^ and the formation of disciples. A later publication reported that many surgeons applied their new expertise in private practice, while few remained in public reconstructive services, highlighting the importance of educating not only fellows but also including oncoplasty in the curriculum of all breast surgeons^23^.

In Brazil, in 2012, the first report of an oncoplasty training course for senior mastologists was observed, showing positive results for attendees in 2009 and 2010^20^. Over time, other courses were created^27^. Table 1 synthesizes information about the courses up to the present date. The model positively impacted clinical practice^20,21^. Among the continuous courses held in Brazil, the number of classes varied from 200 h to 540 h. Theoretical discussions were associated with clinical practice, in which multiple simultaneous surgical rooms, various types of surgery, and bilateral surgeries helped enhance surgical skills. From the available information, the shortest course was 200 h, and it yielded satisfactory^21^ results. As a criterion for participation in the courses, breast surgeons should be board certified. Initially, student selection was based on decentralization, academic relevance, and the potential to train new surgeons, aiming to maximize the impact of the training.

This hands-on course model in Brazil has inspired similar courses in other countries, such as Argentina and Peru, with live surgeries conducted in both face-to-face and virtual formats but in a more concise format with less workload and fewer surgeries at each meeting. Similar hands-on courses with live surgery sessions lasting 1 or 2 days are being offered in other countries, including Colombia, Mexico, Spain, and Germany. International collaboration can benefit developed countries since low- and middle-income countries have shown astonishing ease in training breast specialists and developing new surgical techniques^24^.

There is no predefined duration for training junior surgeons^28^. In Brazil, since 2014, there have been 42 fellows in OPS, all breast surgeons, who were trained for one year by other breast oncoplastic surgeons. From 2009 to 2023, an estimated 452 seniors and 42 juniors were trained, representing approximately 30% of mastologists in Brazil.

Internationally, various basic training courses for breast surgeons exist, which may follow training in general surgery, oncological surgery, or gynecology. OPS training is considered a secondary surgical skill^38^ In Brazil, breast cancer surgeries are performed by mastologists and oncological surgeons. Mastology is a two-year specialty, with initial two- or three-year training in general surgery or gynecology^39^. Oncoplasty training was initially secondary to general mastology training but is gradually being integrated into residency programs, a process that will take more time to consolidate.

Evaluating the training of the breast surgeon in Spain^29^, OPS is a part of the competencies required for breast surgeon training, although publications on this subject were not observed. In Spain, there has been a traditional course for several years that includes lectures, video presentations, and surgeries on pigs^19^, attracting attendees from various countries. In Brazil, oncoplasty is integrated into the training program for mastology residents. By the end of the first year, residents should have mastered level I oncoplastic procedures, and by the end of the second year, they should have attained competency in level II and breast reconstructions^39^. Medical residency programs must adapt to these guidelines. A survey conducted among mastology residents from 2015 to 2016 found that 60% of residents had training in oncoplasty throughout their residency. In breast units where mastologists perform oncoplasty, residents are better prepared to perform oncoplasty and reconstruction techniques^30^.

In Brazil, the Brazilian Society of Mastology offers an official oncoplasty course (https://oncoplasticsurgerycourse.com/en) with live broadcasting of 100 reconstructive procedures, held over two days per month for 11 months. The course provides simultaneous translation in Portuguese, English, and Spanish for breast surgeons, surgical oncologists, and plastic surgeons. In the United States, an online course with home study tools and simulator models organized by the Oncoplastic Surgery Society is available (https://oncoplasticmd.org). In India, the International School of Oncoplasty offers theoretical courses, simulator courses, and a 2-year master's program in oncoplastic surgery (www.breastoncoplasty.org). The European Institute of Oncology in Italy organizes a two-day course with live broadcasting of reconstructive procedures once a year (www.ieo-oncoplastic.com). The American College of Surgeons is planning a course on oncoplastic breast surgery (https://learning.facs.org/content/oncoplastic-breast-surgery). However, there are no publications reporting their outcomes, and there is no standardization of methods and types of procedures.

Additional measures that should be taken include continuing education through the inclusion of an oncoplasty section in national and regional congresses or events, as well as hosting specific oncoplasty congresses. The Brazilian Congress of Mastology and São Paulo Mastology Journey dedicate a period to discussing oncoplasty, offering 4 h of content for about 1000 mastologists each year. The Brazilian Journey of Oncoplasty, initiated by the Brazilian Society of Mastology in 2012, has allowed mastologists to discuss the topic for over a decade, with an average annual participation of more than 300 attendees.

From future perspectives, there is a need to conduct more studies that evaluate learning curves in training breast surgeons and the impact of different methodologies. Additionally, there is a need to increase the number of training centers with associated publications and study trend curves. Oncoplasty is becoming increasingly integrated into daily practice due to increase in both the learning curve and the rate of BCT secondary to neoadjuvant chemotherapy. Reconstructions, initially performed through myocutaneous flaps, have transitioned to implant-based techniques, significantly simplifying the procedures. OPS depends on training, and the more training one receives, the broader the range of potential indications and availability. This is evident in the increasing number of publications related to oncoplasty, in which breast surgeons play a significant role^25^. There is gradually an increase in the number of reconstructions in the public health system in Brazil^40^. To further improve results, it is essential to focus on various aspects such as residency programs, fellowships, oncoplasty training centers, and continuing education. These efforts will ultimately lead to better treatment for breast cancer patients, who are vulnerable and deserving of high-quality care, thereby justifying the need for educating breast surgeons.
